# Seasonal Variation in the Hepatoproteome of the Dehydrationand Freeze-Tolerant Wood Frog, *Rana sylvatica*

**DOI:** 10.3390/ijms12128406

**Published:** 2011-11-29

**Authors:** Andor J. Kiss, Timothy J. Muir, Richard E. Lee, Jon P. Costanzo

**Affiliations:** 1 Laboratory for Ecophysiological Cryobiology, Department of Zoology, Miami University, Oxford, OH 45056, USA; E-Mails: timmuir@augustana.edu (T.J.M.); leere@muohio.edu (R.E.L.); costanjp@muohio.edu (J.P.C.); 2 Center for Bioinformatics and Functional Genomics, Miami University, Oxford, OH 45056, USA; 3 Department of Biology, Augustana College, Rock Island, IL 61201, USA

**Keywords:** anuran, hibernation, LC-MS/MS, iTRAQ™, metabolism, seasonal variation

## Abstract

Winter’s advent invokes physiological adjustments that permit temperate ectotherms to cope with stresses such as food shortage, water deprivation, hypoxia, and hypothermia. We used liquid chromatography (LC) in combination with tandem mass spectrometry (MS/MS) quantitative isobaric (iTRAQ™) peptide mapping to assess variation in the abundance of hepatic proteins in summer- and winter-acclimatized wood frogs (*Rana sylvatica*), a northerly-distributed species that tolerates extreme dehydration and tissue freezing during hibernation. Thirty-three unique proteins exhibited strong seasonal lability. Livers of winter frogs had relatively high levels of proteins involved in cytoprotection, including heat-shock proteins and an antioxidant, and a reduced abundance of proteins involved in cell proliferation, protein synthesis, and mitochondrial function. They also exhibited altered levels of certain metabolic enzymes that participate in the biochemical reorganization associated with aphagia and reliance on energy reserves, as well as the freezing mobilization and post-thaw recovery of glucose, an important cryoprotective solute in freezing adaptation.

## 1. Introduction

Ectotherms inhabiting temperate regions must cope with seasonal variation in resources and environmental conditions that challenge their survival. Amphibians that hibernate terrestrially must contend with prolonged aphagia, limited availability of environmental water, impairment of gas exchange, and potentially extreme hypothermia that may cause tissues to freeze. Physiological adaptation to these stresses commonly requires changes in gene expression that tune the quantities of various proteins to maintain cellular integrity and regulate metabolic processes. Such alterations become conspicuous in the proteome, and quantification of these changes can provide important insights into the adaptive mechanisms functioning at multiple levels of biological organization. In this profiling study, we used liquid chromatography (LC) in combination with tandem mass spectrometry (MS/MS) quantitative isobaric (iTRAQ™) peptide mapping to investigate seasonal variation in the abundance of proteins in liver of the wood frog, *Rana sylvatica*. While overwintering beneath duff in upland forests, these frogs survive many months without feeding and must endure dehydration and the freezing of up to 65–70% of their body water [1]. Our aim in this project was to gain insights into the macromolecular responses contributing to winter survival in this freeze-tolerant species.

## 2. Results and Discussion

Considering that the genome for *R. sylvatica* has not been published, we used a highly conservative criterion for identifying proteins at the 95% level of confidence. A total of 1406 distinct peptides were identified from 1860 spectra, and the peptides were matched to an initial set of 340 proteins. We discarded any protein whose iTRAQ™ ratio (a relative measure of abundance) varied by ≥20% between the two members comprising each seasonal group. Of the remaining 67 proteins, we considered further only those proteins that differed in abundance between summer and winter groups by at least 20%, the combined maximal error rate of the iTRAQ™ technique used in conjunction with MALDI ToF/ToF [2]. Identification of the remaining 37 proteins was aided by the diverse taxonomic representation in the NCBInr database (including several species of amphibians, such as *Xenopus* spp., for which a sequenced genome is available) and by the evolutionary conservation of many of the proteins of interest, such as those of the major metabolic pathways [3]. Nevertheless, because the functions of homologous proteins are not necessarily identical, we deem our conclusions tentative until confirmed by more targeted methodologies. In four cases, distinct peptide fragments from a given protein were matched to two database entries; consequently, we discarded the duplicate exhibiting the lower of the two ratios.

Of the remaining 33 unique proteins exhibiting marked seasonal variation, most (19; 58%) were reduced in abundance in winter relative to summer. This group included mitochondrial enzymes as well as proteins involved in cell growth and proliferation (Table 1). The smaller set of up-regulated proteins contained relatively few metabolic enzymes and was dominated by proteins important for preserving cellular integrity and function (Table 2).

**Figure 1:**
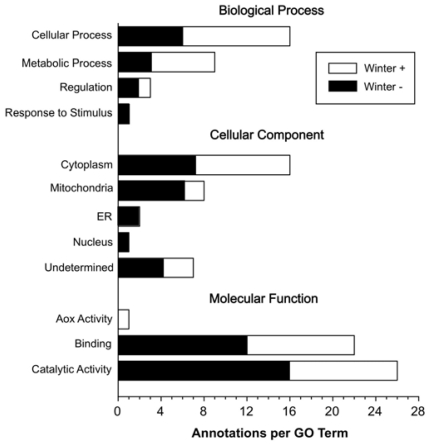
Gene ontology (GO) annotation by STRAP analysis of 33 differentially-expressed proteins from livers of summer- and winter-acclimatized *Rana sylvatica*. Filled and unfilled segments of each bar indicate the numbers of proteins that were downregulated and upregulated, respectively, in winter frogs.

Gene ontology (GO) analysis using the computer program STRAP [4] revealed that the seasonally labile proteins were well represented in the cellular and metabolic process bins of the biological process domain (Figure 1). Most of these proteins were localized to the cytoplasm or mitochondrion, although the latter’s complement primarily exhibited reduced abundance in winter. Proteins having catalytic and binding activity showed either increased or decreased expression in winter frogs, whereas ER proteins were only reduced (Figure 1).

The caveat that changes in protein expression do not necessarily alter the functional processes in which they participate notwithstanding, seasonal dynamics of hepatic proteins are interpretable in the context of the unique physiological demands and stresses imposed on *R. sylvatica* during winter. Downsizing the machinery of cell proliferation and somatic growth are hallmark responses to dormancy in diverse taxa [5] and, accordingly, observed alterations in winter frogs seemingly support a regulated reduction in biosynthetic functions. Diminished protein synthesis is reflected in the lowered abundance of regulatory proteins, including mRNA transport regulator and elongation factor 1-α (EF1-α), a central component of the active translation and elongation machinery. *R. sylvatica* up-regulates translation of the ribosomal protein L7 gene, whose product is thought to inhibit translation, during cold acclimation [6].

Several of the seasonally labile proteins have roles in preserving cellular and macromolecular integrity in the face of low temperature, dehydration, and limited energy flow. For example, the winter up-regulation of actin-related protein, an important regulator of filament polymerization, could offset the depolymerizing effect of cold. Winter frogs had higher levels of certain heat-shock proteins (Hsps), members of a family of molecular chaperones that assist in the folding and translocation of intracellular proteins and, under stress, refold and inhibit the aggregation of denatured proteins. Induction of Hsp synthesis preparatory to winter or in cold acclimation is seen in diverse taxa, suggesting that these chaperones play a general role in stabilizing proteins at low temperature [7]. Both the inducible Hsp70 and its cognate counterpart, Hsc70, were among the proteins most strongly up-regulated in winter frogs. This response could help preserve the extant pool of functional proteins at a time when protein turnover is curtailed; it may also contribute to freezing survival, since *Hsp* gene expression in *R. sylvatica* is not further increased during freezing [8].

Winter frogs exhibited not a rise, but rather a decrease, in the level of BiP (=GRP78), another chaperonin of the Hsp70 family. This ER resident exhibits variable and tissue-specific expression in dormancy, but decreases markedly in the liver of some hibernators [5]. Maintaining a relatively low abundance of this protein, an important stress sensor, in winter potentially enhances sensitivity of the unfolded protein response (UPR), a conserved cellular pathway that mitigates stress-induced proliferation of unfolded proteins [5,9].

Various proteins with antioxidation properties commonly are seen to increase during chilling or dormancy [7] and, accordingly, our winter frogs had increased levels of peroxiredoxin, a member of a ubiquitous family of sulfhydryl-linked antioxidants. Besides its role in reducing organic hydroperoxides, this protein is transformed under oxidative stress, such as occurs when *R. sylvatica* freezes, to a high-molecular-mass complex that exhibits molecular chaperone activity [10]. GAPDH, which was also up-regulated in winter frogs, has several non-glycolytic functions, including DNA repair and antioxidation [11].

Winter frogs apparently altered metabolic processes to accommodate a reduced energy demand and increased use of energy reserves. Among temperate amphibians, fat-body lipids are consumed chiefly in the pre-hibernal period [12] and, accordingly, *R. sylvatica* has little adipose remaining by the time our winter frogs were sampled [13]. Changes in certain enzymes of lipid metabolism that curtail lipid use in hibernating frogs included a reduction in acetyl-CoA acetyltransferase 1 (ACAT1), which in liver plays an essential role in the degradation of fats and proteins, and long-chain acyl-CoA dehydrogenase. The latter, which requires FADH_2_ as a co-factor and thus can function in redox balancing in both β-oxidation and electron transport, contributes to the oxidation of C12–C18 fatty acids, which are key components of the triacylglycerides comprising storage lipids. On the other hand, winter frogs apparently up-regulated levels of hydroxyacyl-CoA dehydrogenase, a mitochondrial oxidoreductase that catalyzes the β-oxidation of medium chain-length fatty acids, which, derived primarily from sources other than storage fats, enter the mitochondria without need of the carnitine transport system and undergo preferential oxidation. Increased levels of the latter enzyme in liver could help aphagic frogs produce energy and synthesize ketone bodies, an important source of energy in brain, muscle, and other tissues during fasting. The presumed increase in glycolytic activity (see below) could also contribute to ketogenesis in hibernating frogs.

Enzymes mediating the interconversion and catabolism of amino acids, as well as nitrogenous waste formation, are commonly down-regulated in winter dormancy in various species [5]. This seems also true of *R. sylvatica*, as a reduction in amino acid and urea metabolism in winter frogs was evidenced by a lowered abundance of glutamic-oxaloacetic transaminase 1, alanine-glyoxylate transaminase, histidase, acetyl-CoA acetyltransferase 1, glutamate dehydrogenase 1, homogentisate 1,2-dioxygenase, and, rather strongly, carbamoyl phosphate synthase 1 (CPS I). Immunoblotting studies also indicate that the latter enzyme, which controls the rate-limiting step in the ornithine cycle, is down-regulated in winter *R. sylvatica*; however, its activity is nevertheless maintained, apparently though regulation via posttranslational modification and/or feedback inhibition [14]. Preserving a heightened activity of CPS I throughout autumn and winter presumably allows this species to accumulate urea as a cryoprotectant [1] and metabolic depressant [15]. Among other temperate frogs, urea synthesis rate is markedly lower during fall/winter as compared to spring/summer [12].

Metabolic reorganization in the hibernating phenotype of *R. sylvatica* also involved marked changes in quantities of key glucoregulatory enzymes. Control of carbohydrate metabolism in hibernating frogs is complex in that cold acclimation and starvation can independently modify activities of glycolytic and gluconeogenic enzymes; however, fasting typically reduces flux through pathways of protein catabolism and glucose synthesis [12]. The coupled increase in pyruvate kinase and decrease in phosphoenolpyruvate carboxykinase (PEPCK) in winter indeed suggest that hibernating *R. sylvatica* undergo a stimulation of glycolysis and diminished reliance on gluconeogenesis. This interpretation concurs with a report that, in bullfrog (*R. catesbeiana*) liver, the activity of PEPCK in winter is only about 20% of that measured in summer, when gluconeogenesis is the predominant pathway for the large dietary influx of amino acids [16]. The increased abundance of pyruvate kinase probably bears little on the ability of *R. sylvatica* to accumulate glucose during freezing episodes, as tissue freezing imposes a block on glycolysis by inhibiting phosphofructokinase [17].

In hibernating frogs, the hepatic glycogen reserve is an important source of glucose that provides a metabolic fuel and, in freeze-tolerant species, a cryoprotectant that enhances freezing survival [12]. Accordingly, activity of glycogen phosphorylase is relatively high during winter in both freeze-tolerant and freeze-intolerant frogs [18–20]. The greater abundance of this enzyme in our winter frogs undoubtedly increases their capacity to rapidly mobilize glucose in response to tissue freezing. After thawing, *R. sylvatica* quickly replenishes the hepatic glycogen reserve from the copious free glucose, and this process could be expedited through the observed rise in protein phosphatase-1, a serine/threonine-specific phosphatase that regulates a host of cellular functions, including glycogen metabolism. Under insulin activation, this enzyme dephosphorylates both glycogen synthase (activating reaction) and glycogen phosphorylase (deactivating reaction), thereby promoting glycogen deposition. Rebuilding the reserve would be further aided by the up-regulation of the glycosyltransferase observed in winter frogs.

Seasonal changes in the metabolic organization of *R. sylvatica* involved not only certain enzymes of glycolysis, gluconeogenesis, and amino acid and lipid metabolism, but also those that drive the TCA cycle. Reduced abundance in winter frogs of key cataplerotic enzymes, including PEPCK, aspartate aminotransferase, and glutamate dehydrogenase, a chief enzyme of the malate-aspartate shuttle, presumably reflects a limited need for gluconeogenic flux from amino acid metabolism. Down-regulation of these enzymes, coupled with the observed increase in pyruvate carboxylase, a primary anaplerotic enzyme, potentially leads to accrual of oxaloacetate (OAA) in the mitochondria. This intermediate could be converted to citrate, which then enters the TCA cycle via (winter up-regulated) aconitase; ultimately, however, OAA would be regenerated. Speculation suggests that a putative accrual of this intermediate could contribute to antioxidation in hibernating frogs, as OAA is a known inhibitor of complex II [21], a major contributor to superoxide formation via reverse electron transfer to complex I [22]. Muller *et al*. [22] suggested that OAA inhibition of complex II is an adaptive mechanism to minimize superoxide formation, and this effect may partially explain the extraordinarily low level of oxidative damage found in frozen/thawed *R. sylvatica* [23]. Further investigation into possible accumulation of OAA and its potential role in antioxidation in this and other freeze-tolerant species may prove rewarding.

## 3. Experimental Section

Male *R. sylvatica* were collected in February from breeding pools in southern Ohio and subsequently kept in an outdoor enclosure that provided naturalistic thermal, hydric, and photic regimes [1]. They were fed crickets thrice weekly, but also consumed other invertebrates incidentally. In August, two “summer” frogs were transferred to our laboratory and kept in a moist terrarium at 21 °C (12:12, L:D) for 3 day before their livers were removed. Other individuals were gathered in early November, soon after they had ceased feeding and entered hibernacula. Two of these “winter” frogs, were kept in a moist terrarium at 4 °C in total darkness (conditions simulating natural hibernation) until they were sampled for liver tissue, in early February.

Frogs were euthanized by double-pithing and rapidly dissected, and a portion of the medial hepatic lobe was excised, rinsed in phosphate-buffered saline (PBS), blotted dry, and frozen in liquid nitrogen. Following brief storage at −80 °C, tissue samples were weighed, homogenized under liquid nitrogen, suspended in PBS, and assayed for soluble protein (Bradford; BioRad). Homogenates containing ~110 mg·mL^−1^ soluble protein were briefly stored at −80 °C before being shipped on dry ice to Michigan Proteome Consortium, University of Michigan (Ann Arbor, MI, USA), for proteomic analysis. Institutional Animal Care and Use Committee (IACUC) of Miami University approved the experimental protocols.

Protein abundance was evaluated using isobaric tag for relative and absolute quantitation (iTRAQ™) reagent in an assay designed to characterize protein mixtures in complex biological systems [24]. This high-throughput workflow labels samples with multiple, independent reagents of the same mass that, upon fragmentation in MS/MS, yield unique reporter ions that allow peptide quantification. Liver homogenates were conjugated to 4-Plex iTRAQ™ reagent and then separated by 2D LC Multidimensional Protein Identification Technology (MudPIT) [25]. Cysteines were alkylated using MMTS prior to analysis by MS/MS on a 4800 MALDI ToF/ToF instrument (Applied Biosystems, Inc.). Samples were prepared and analyzed by laboratory staff under the supervision of Philip C. Andrews, Director, Michigan Proteome Consortium, under contractual agreement.

Peptide spectra were analyzed using ProteinPilot™ 2.01 software (Applied Biosystems, Inc.), implementing the Paragon™ and Pro Group™ algorithms, and identified by matching the reported sequences to proteins culled from the NCBInr database (GenBank/NCBI). This software requires that at least one unique peptide spectral signature be assigned to a given protein, thus removing it from the available pool of spectra, before rendering a positive identification. This approach precludes making multiple assignments of peptide spectra to different proteins, thus providing the currently most defensible criteria for accurate protein identification [26].

## Figures and Tables

**Table 1:** Proteins identified from extracts of liver from *Rana sylvatica* that were more abundant in summer than in winter.

GI	Protein	AR 1	Species	Function
129730	protein disulfide isomerase precursor	2.34	*Oryctolagus cuniculus*	protein folding
9755362	acetaldehyde dehydrogenase	2.28	*Mus musculus*	alcohol metabolism
85719973	peptidylprolyl isomerase B	2.22	*Ictalurus punctatus*	protein folding
57087309	mRNA transport regulator 3	1.74	*Canis familiaris*	protein synthesis
47498070	aspartate aminotransferase 1	1.68	*Xenopus tropicalis*	amino acid metabolism
146415164	RNA recognition motif (hypothetical)	1.65	*Pichia guilliermondii*	protein synthesis
5921957	carbamoyl-phosphate synthase I	1.53	*Rana catesbeiana*	urea metabolism
148226795	binding immunoglobulin protein (BiP)	1.51	*Xenopus laevis*	protein folding/stress
121594245	protein DUF891	1.51	*Acidovorax* sp.	unknown
47210694	glutamate dehydrogenase 1	1.49	*Tetraodon nigroviridis*	nitrogen metabolism
45383354	histidine ammonia-lyase	1.38	*Gallus gallus*	amino acid metabolism
124266729	acetyl-CoA C-acetyltransferase 1	1.34	*Methylibium petroleiphilum*	protein/lipid metabolism
58332740	aconitase 2	1.33	*Xenopus tropicalis*	TCA cycle
148230238	homogentisate 1,2-dioxygenase	1.32	*Xenopus laevis*	amino acid metabolism
126632707	long-chain acyl-CoA dehydrogenase	1.26	*Danio rerio*	fatty acid metabolism
50417404	alanine-glyoxylate aminotransferase	1.24	*Xenopus laevis*	amino acid metabolism
56377788	elongation factor 1-α	1.22	*Pelodiscus sinensis*	protein synthesis
89886140	phosphoenolpyruvate carboxykinase 1	1.21	*Xenopus tropicalis*	gluconeogenesis

1 AR, abundance ratio: protein abundance in summer frogs relative to that in winter frogs.

**Table 2:** Proteins identified from extracts of liver from *Rana sylvatica* that were more abundant in winter than in summer.

GI	Protein	AR 1	Species	Function
147901600	glycogen phosphorylase	2.04	*Xenopus laevis*	glycogen catabolism
145545139	protein kinase C	1.72	*Paramecium tetraurelia*	signal transduction
148227386	pyruvate carboxylase, gene 1	1.67	*Xenopus laevis*	gluconeogenesis
134254218	pyruvate carboxylase, gene 2	1.51	*Xenopus tropicalis*	gluconeogenesis
118083730	1,4-α-glucan branching enzyme	1.42	*Gallus gallus*	glycogen synthesis
63146078	heat shock protein 70 (hsp70)	1.34	*Oxyuranus scutellatus*	stress response
2196882	heat shock cognate protein 70 (hsc70)	1.32	*Pleurodeles waltl*	protein folding
148225037	pyruvate kinase type M2	1.32	*Xenopus laevis*	glycolysis
147902026	peroxiredoxin 6	1.30	*Xenopus laevis*	antioxidation
91084329	protein phosphatase-1 α	1.29	*Tribolium castaneum*	glycogen metabolism
148234835	β-ureidopropionase	1.27	*Xenopus laevis*	amino acid metabolism
54020777	hydroxyacyl-CoA dehydrogenase	1.24	*Xenopus tropicalis*	fatty acid metabolism
148224534	Arp2/3, subunit 2	1.24	*Xenopus laevis*	actin polymerization
148223127	glyceraldehyde-3-phosphate dehydrogenase	1.21	*Xenopus laevis*	glycolysis; non-metabolic processes

1 AR, abundance ratio: abundance ratio: protein abundance in winter frogs relative to that in summer frogs.
